# Malic enzyme and glucose 6-phosphate dehydrogenase gene expression increases in rat liver cirrhogenesis.

**DOI:** 10.1038/bjc.1997.85

**Published:** 1997

**Authors:** N. Sanz, C. Díez-Fernández, A. M. Valverde, M. Lorenzo, M. Benito, M. Cascales

**Affiliations:** Instituto de Bioquímica (CSIC-UCM), Facultad de Farmacia, Madrid, Spain.

## Abstract

**Images:**


*British Journal of Cancer* (1997) **75**(4), 487–492
© 1997 Cancer Research Campaign

### Malic enzyme and glucose 6-phosphate dehydrogenase gene expression increases in rat liver cirrhogenesis

**N Sanz, C Díez-Fernández, AM Valverde, M Lorenzo, M Benito and M Cascales**

Instituto de Bioquímica (CSIC-UCM), Facultad de Farmacia, Plaza de Ramón y Cajal sn, 28040 Madrid, Spain

**Summary** The cirrhogenic ability of thioacetamide has been used to induce a model of chronic generalized liver disease that resembles the preneoplastic state of human fibrosis. Malic enzyme (ME) and glucose-6-phosphate dehydrogenase (G6PDH) are two cytosolic NADPH-generating enzymes; their activities significantly increased in liver when macronodular cirrhosis was induced by long-term thioacetamide administration to rats. The progressive increase in G6PDH and ME activities during the cirrhogenic process is parallel to the induction in gene expression of both enzymes detected by the increase in their mRNAs. These data indicate that NADPH-consuming mechanisms such as the microsomal oxidizing system and the maintenance of the cell redox state could be involved. A relationship between the extent of G6PD and ME gene expression and oxidative stress generated by the oxidative metabolism of thioacetamide is proposed as the hepatic concentration of malondialdehyde, a metabolite derived from lipid peroxidation, underwent a progressive and significant enhancement during thioacetamide-induced cirrhogenesis. These results led us to suggest that the enhanced activities of G6PDH and ME might be related to microsomal mechanisms of detoxification as well as to the maintenance of the cellular redox state. Furthermore, the noticeable increase in the hepatocyte population involved in DNA replication parallel to G6PDH activity suggests that G6PDH, through ribose-5-phosphate, might also be involved in the processes of DNA synthesis and repair.

**Keywords:** NADPH-generating enzyme; oxidative stress; cirrhogenesis; gene expression

Long-term administration of thioacetamide to rats induces cirrhosis, tumours and hepatocarcinoma (Tsukamoto et al, 1990). Attempts have been made to detect the irreversible stage in the sequence of events – damage and inflammation → cholestatic cirrhosis → hepatocellular carcinoma – as it is well known that cirrhotic livers, through the emergence of adenomatous hyperplastic nodules, are considered to be precancerous lesions (Pitot, 1990). Hepatic fibrosis is related to inflammation of the liver and may eventually constitute an irreversible process with diffuse parenchymal nodular transformation, chronic cholestasis and cirrhosis (Desmet, 1992). This model of hepatic cirrhosis mimics the pathological human hepatic fibrosis and can be considered as a preneoplastic liver disease (Farber, 1990).

Glucose-6-phosphate dehydrogenase (G6PDH) (EC 1.1.1.49) is a key enzyme that catalyses the first reaction in the pentose phosphate pathway, leading to the production of ribose 5-phosphate for nucleic acid synthesis and reducing power in the form of NADPH for reductive biosynthesis. Hepatic G6PDH is strongly modulated in response to external stimuli, such as hormones, growth factors, nutrients and oxidative stress (Katsurada et al, 1989; Iritani, 1992; Kletzien and Berdainer, 1993; Tomita et al, 1993). Malic enzyme (ME) (EC 1.1.1.40), another NADPH-generating enzyme that catalyses the oxidative decarboxylation of L-malate to yield carbon dioxide, pyruvate and NADPH, is considered to be a lipogenic enzyme whose activity correlates with de novo fatty acid synthesis (Karsurada et al, 1987; García-Jiménez et al, 1994).

*Received 17 April 1996*
*Revised 4 September 1996*
*Accepted 12 September 1996*

*Correspondence to:* M Cascales

Reducing equivalents generated by the NADP-dependent systems are involved in three main cellular mechanisms: lipogenesis, maintenance of the cell redox state and the microsomal reactions of drug detoxification. The major sources of cytosolic NADPH are G6PDH and ME (Karsurada et al, 1987; García-Jiménez et al, 1994), and it has been thought that the activities of these two enzymes are mainly involved in de novo fatty acid synthesis (Iritani, 1992; Tomita et al, 1993). However, the noticeable and progressive increase in these two enzyme activities in thioacetamide-induced macronodular cirrhotic liver (Cerdán et al, 1981; Martín-Sanz et al, 1989*a*; Nozu et al, 1992) was not parallel either to the in vivo lipogenesis or to other enzyme activities involved in fatty acid synthesis, such as ATP citrate lyase, acetyl-CoA carboxylase and fatty acid synthetase, which decrease significantly during this cirrhogenic process (Martín-Sanz et al, 1989*a*). Accordingly, the excess of NADPH produced by the enhanced activities of G6PDH and ME could be owing to the requirement of the microsomal mono-oxygenase NADPH-dependent processes (Kletzien et al, 1994) and to the maintenance of the redox state of the cell.

As the generation of reactive oxygen species and reactive metabolites by the microsomal oxidizing system is enhanced by NADPH (Cramer et al, 1993), the relationship between NADPH-generating systems (G6PDH and ME) and the oxidative stress has been studied in a macronodular cirrhotic liver induced by chronic administration of thioacetamide to rats. The induction of G6PDH activity by agents that induce oxidant stress and lipoperoxidation (Cascales et al, 1991; Muller et al, 1991; Bautista and Spitzer, 1992) led us to consider that the G6PDH gene responds to the oxidative attack providing protection in the form of NADPH to maintain the cellular redox state (Cramer et al, 1993; Kletzien et al, 1994). G6PDH activity, through the generation of ribose 5-phosphate, is also involved in nucleic acid synthesis and repair as when hepatocyte growth is stimulated, the expression of this enzyme is also stimulated, favouring NADPH and pentose phosphate generation (Yoshimoto et al, 1983; Molero et al, 1994).

NADPH redox systems play an important role in activating and detoxifying chemical carcinogens (Cramer et al, 1993; Kletzien et al, 1994), and G6PDH also plays a role in DNA replication and repair (Yoshimoto et al, 1983; Molero et al, 1994). The purpose of the present study was to determine G6PDH and ME activities and gene expression at the mRNA level during the macronodular hepatic cirrhogenesis induced by long-term thioacetamide administration to rats. The results obtained in the present investigation show that the noticeable increases in the activities of both G6PDH and ME enzymes are due to an enhancement in their respective gene expression. These values are parallel to the extent of increase in lipid peroxidation owing to oxidative stress, according to the hepatic concentration of malondialdehyde and also to the increased rate of cell proliferation.

### MATERIALS AND METHODS

### Chemicals

Enzymes were obtained from Boehringer Mannheim (Mannheim, Germany). Substrates and coenzymes were from Sigma (St Louis, MO, USA). Standard analytical grade laboratory reagents were obtained from Merck (Darmstadt, Germany). [$\alpha$-$^{32}$P]dCTP (3000 Ci mmol$^{-1}$) and multiprimer DNA-labelling system kit were purchased from Amersham. Rat G6PDH cDNA (pKs-G6PDH) and rat ME cDNA (pME6) were kindly provided by Dr Ho and Dr Nikodem (Magnuson and Nikodem, 1983; Ho et al, 1988). Cycle-Test DNA reagent kit was from Becton Dickinson (San José, CA, USA).

### Animals and treatment

Two-month-old adult male Wistar rats (180–220 g), supplied with food and water ad libitum and exposed to a 12-h light–dark cycle, were given intraperitoneally continuous doses, three times every week, of thioacetamide (2.66 mmol kg$^{-1}$ body weight) freshly dissolved in 0.9% sodium chloride. To equilibrate the nutritional intake and the conditions, a parallel group of rats was supplied the same feeding as that consumed by the thioacetamide-treated rats on the previous day and were given intraperitoneal injections of 0.5 ml of 0.9% sodium chloride. The nutritional status of the animals did not essentially change as the daily intake/body weight ratio remained practically unchanged. Fetuses were obtained from pregnant albino Wistar rats (300–350 g) by caesarian section in the morning of the 22nd day of gestation. Gestational state was checked according to the standard criteria used in previous studies (Cascales et al, 1992). Each experiment was repeated four times and followed the international criteria for the use and care of experimental animals in research. Standards and procedures outlined in the *Guide for the Care and Use of Laboratory Animals* prepared by the National Academy of Sciences and published by the National Institute of Health (NIH publication no. 80–83, revised 1985) were observed.

### Processing of the samples

To follow the time course of the changes induced by the long-term treatment with thioacetamide, samples were obtained at 0, 7, 15, 30, 45, 60, 90, 120, 150 and 180 days. Rats were cervically dislocated and pieces of approximately 500 mg of liver were quickly freeze clamped in situ using stainless-steel tongs cooled in liquid nitrogen and then removed and placed in liquid nitrogen until processed for Northern blot analysis. A 1-g sample of fresh liver was homogenized in ice cold solution of 0.25 M sucrose with a loose-fitting Teflon glass Potter-Elvehjem homogenizer to make a 20% (w/v) homogenate; the homogenate was centrifuged at 105 000 ***g*** for 45 min at 4°C, and the supernatant (soluble fraction) was dialyzed for 2 h (Díez-Fernández et al, 1993).

### Histopathological study

For histopathological investigations, pieces of liver obtained from rats at 6 months (180 days) of continuous thioacetamide exposure were fixed in 10% formaldehyde, embedded in paraffin, sectioned (5 μm) and stained with Masson's trichrome (Luna, 1968).

### Isolation of hepatocytes and flow cytometry analysis

Hepatocytes were isolated according to the classic collagenase perfusion method. Fetal hepatocytes were obtained from 22-day fetuses after digestion of the fetal liver with collagenase (Martín-Sanz et al, 1989). Cell viability, determined by trypan blue exclusion, was greater than 90%.

For the analysis of DNA content, $1 \times 10^6$ viable cells were stained with propidium iodide following the multistep procedure of Vindelov et al (1983). The emitted fluorescence of the DNA–propidium iodide complex was assayed in a FACScan flow cytometer (Becton-Dickinson). A double discriminator module was used to distinguish between signals coming from a single nucleus and those produced by nuclear aggregation. Data were analysed by evaluating single-nucleus inputs ($10^4$ nuclei per assay).

### Enzyme activities and metabolite assays

Enzymatic determinations were carried out in the soluble fraction of liver homogenates in the optimal conditions of pH and temperature and with substrates and cofactors at saturation. Glucose-6-phosphate dehydrogenase was determined spectrophotometrically at 340 nm in the presence of glucose-6-phosphate and NADP (Deutsch, 1987), and malic enzyme activity was assayed in the same fraction in the presence of malate and NADP by the method already described (Outlaw and Springer, 1987). Malondialdehyde was determined in liver extracts by homogenizing the samples in three volumes of 0.1% trichloroacetic acid and centrifugation at 15 000 ***g*** for 10 min. Aliquots of the supernatant were added to thiobarbituric acid reagent (5% thiobarbituric acid in 20% trichloroacetic acid) and heated at 90°C for 15 min. Samples were centrifuged and the absorbance was measured in the supernatant at 535 nm. The results were expressed as nanomoles per gram of liver as previously described (Niehaus et al, 1969). Proteins were evaluated by the method of Bradford (1976).

### Northern blot analysis

A 50-mg sample of liver was lysed with guanidinium thiocyanate–phenol reagent for RNA isolation (Chomezynski and Sacchi, 1987). Total cellular RNA (20 μg) was submitted to Northern blot analysis, being electrophoresed on 0.9% agarose gels containing 0.66 M formaldehyde, transferred to GeneScreenTM membranes (New England Nuclear Research Products, Boston, MA, USA) using a VacuGene Blotting apparatus (LKB, Pharmacia,

 © Cancer Research Campaign 1997

Figure 1 Histopathological study of rats liver after 180 days of long-term thioacetamide treatment. Liver slices of 5 μm were obtained from a rat injected with thioacetamide (2.66 mmol $kg^{-1}$) three times per week for 180 days and stained with Masson's trichrome (Luna, 1968). Fibrous septa of collagen develops from two close portal terminals with some haemorrhagic zones. Nodules of different sizes can also be observed, inside which fibrous septa and proliferative ductular cells can be detected. Hepatocytes inside the nodule are swollen and with hypertrophic nucleoli (bar = 50 μm)

Figure 2 Time course of the levels of malondialdehyde in rat liver extracts during the long-term thioacetamide intoxication. The values are expressed as nmol g liver$^{-1}$ and are the mean ± s.d. of four experimental observations. ● —— ●, control; ▲ - - - ▲, thioacetamide. *$P < 0.001$ *vs* control

Upsala, Sweden) and cross-linked to the membranes by UV light. Hybridization was in 0.25 mM sodium phosphate pH 7.2, 0.25 M sodium chloride, 100 μg $ml^{-1}$ denatured salmon sperm DNA, 7% sodium dodecyl sulphate (SDS) and 50% deionized formamide, containing denatured $^{32}$P-labelled cDNA ($10^6$ c.p.m. $ml^{-1}$) for 40 h at 42°C as described previously (Amasino, 1986). cDNA labelling was carried out with [α-$^{32}$P]dCTP to a specific activity of $10^9$ c.p.m. μ$g^{-1}$ of DNA by using a multiprimer DNA-labelling system kit (Amersham, Buckinghamshire, UK). For serial hybridization with different probes, the blots were stripped and rehybridized sequentially as needed in each case. The resulting membranes were subjected to autoradiography for 1–3 days. Relative densities of the hybridization signals were determined by densitometric scanning of the autoradiograms in a laser densitometer (Molecular Dynamics, Sunnyvale, CA, USA). Finally, the filters were hybridized with a 18S rRNA probe for RNA normalization. The analysis of the Northern blot was performed in duplicate from two livers. The variability in the measurement of mRNA, after quantification by scanning densitometry from the filters, was not greater than 15%.

### Statistical analysis

Student's *t*-test was performed for statistical evaluations. The statistical significance has been considered as $P<0.001$.

### RESULTS

Hypertrophic cirrhosis is characterized by the appearance of nodules surrounded by fibrous septa of collagen. In our experiments, macronodular cirrhosis was induced by long-term thioacetamide administration, and the histopathological analysis was performed in liver from a rat after 6 months' (180 days) treatment. Figure 1 shows the morphology of a liver slice stained with Masson's trichrome. Fibrous septa of collagen appear between every two adjacent portal terminals surrounding the hepatic parenchyma. Some haemorrhagic zones can be observed. Nodules of different sizes are responsible for the liver appearance and the hepatocytes inside show hypertrophic nucleoli. These characteristics, typical of the macronodular hypertrophic cirrhosis, resemble human cirrhosis and are included in the process of preneoplastic liver disease. According to Becker (1983) and Tsukamoto et al (1990), following thioacetamide treatment for 6 months the liver shows advanced septal fibrosis and ductal proliferation giving rise to the histological appearance of cirrhosis. The ductal proliferation is one of the first signs of cholangiofibromas and cholangiocarcinomas.

In order to detect whether the hepatic metabolism of thioacetamide, in our experimental conditions, produces oxidative stress and lipid peroxidation, malondialdehyde (MDA) concentration was determined in liver during the cirrhogenic process. Figure 2 shows the concentration of MDA in liver extracts obtained from rats during the long-term thioacetamide treatment. MDA increases progressively, reaching at day 45 of the treatment 2.5 ± 0.2 times ($P<0.001$) the value of the time-matched controls. From this point until the end of the experiment, hepatic concentration of MDA was maintained between 2.5 and 2.8 times above the control. Samples obtained from control rats did not show significant variations with time.

Because the coordinated cellular response to oxidant stress involves a higher requirement of the cell reducing power, the systems that generate reducing equivalents are expected to be enhanced. Accordingly, the time course of G6PDH and ME activities was assayed in the soluble fraction of liver homogenates obtained from rats during long-term thioacetamide treatment. These results are summarized in Figure 3, which shows that the activities of both enzymes, G6PDH and ME, rose progressively and statistically significantly, reaching at the end of the treatment values of 389 ± 29% ($P<0.001$) and 230 ± 20% ($P<0.001$), compared with the chronological controls, respectively. These values show that the increase in G6PDH activity is noticeably higher than that of ME. G6PDH and ME activities in controls showed no significant changes, and no age effects throughout the 6 months of the treatment could be detected. Fetal G6PDH and ME activities were assayed in the soluble fraction of liver homogenates. Fetal G6PDH activity was 64 ± 5.1 nmol $min^{-1}$ mg $protein^{-1}$, which exceeds by 2.5 fold the value for an adult (266 ± 29%, $P<0.001$). However, fetal ME activity was undetectable as this activity is induced in the suckling–weaning transition (Molero et al, 1994).

© Cancer Research Campaign 1997

**Figure 3** Time course of G6PDH and ME enzyme activities in the soluble fraction of rat liver during the cirrhogenesis induced by thioacetamide. The results are expressed as m-units (nmol min$^{-1}$) mg protein$^{-1}$ and are means ± s.d. of four experimental observations. ● —— ●, control; ▲ - - - ▲, thioacetamide. *$P < 0.001$ *vs* control

**Figure 4** Northern blot analysis of G6PD and ME mRNA in liver of rats during long-term thioacetamide treatment. (**A**) A representative Northern blot with a 18S rRNA probe for RNA normalization (N). RNA were isolated from livers from control and TAM-treated rats at 7 (1 and 8), 30 (2 and 9), 60 (3 and 10), 90 (4 and 11), 120 (5 and 12), 150 (6 and 13) and 180 (7 and 14) days respectively. The relative sizes are shown on the right. (**B**) Quantification of the G6PD and ME mRNAs, after correction with 18S rRNA; each TAM value is expressed with respect to its control from two independent experiments. ■, G6PD; □, ME. The data are means and standard deviation. *$P < 0.001$ *vs* control

The increased activities of G6PDH and ME during the thioacetamide-induced cirrhogenesis process described above led us to study the expression of G6PD and ME genes. Figure 4 illustrates the Northern blot analysis of the mRNA content of G6PD and ME. Two isoforms of the malic enzyme mRNA (4.5 kb and 2.7 kb) were found in liver (Dozin et al, 1985). The relative levels of the mRNA were 1:4 respectively. A single form of 2.3 kb was found for G6PD mRNA. Gene expression of these enzymes increases remarkably with time in the cirrhogenic model, the elevation of G6PD being about twice that of ME.

Considering the enhanced activities of G6PDH and its involvement, through the generation of pentose phosphates, in de novo nucleic acid synthesis and cell growth, we studied the extent of DNA replication by means of flow cytometry in isolated hepatocytes obtained during cirrhogenesis. The yield in hepatocytes from thioacetamide-treated liver was approximately 40–60% that of the control liver. Table 1 shows the time course of the hepatocyte population in S-phase of the cell cycle. The values are expressed as the percentage of the cells in the proliferative state. Most of the hepatocytes in normal liver are in a quiescent state, and only about 1% undergo DNA replication. However, when an aggressive attack occurs and cells die, the remaining cells dedifferentiate and divide. These events have been recently studied in acute thioacetamide intoxication (Díez-Fernández et al, 1993), in which cell necrosis is followed by cell proliferation. In the present investigation, in chronic macronodular cirrhosis, replication of DNA

**Table 1** Quantitative analysis of hepatocyte population in S-phase ($S_1 + S_2$) of the cell cycle

| **Treatment (days)** | **Phase S ($S_1 + S_2$)** | |
|---|---|---|
| | **Control** | **Thioacetamide** |
| Fetal[a] | 7.3±1.0 | – |
| 0[b] | 1.1±0.1 | – |
| 7 | 1.5±0.2 | 1.9±0.2 |
| 30 | 1.6±0.2 | 6.2±1.0* |
| 60 | 1.3±0.1 | 4.8±0.5* |
| 90 | 1.3±0.1 | 3.7±0.4* |
| 120 | 1.3±0.1 | 3.9±0.4* |
| 150 | 1.4±0.1 | 3.8±0.4* |
| 180 | 1.6±0.2 | 3.2±0.4* |

The values are expressed as the hepatocyte population (%) in $S_1 + S_2$ phases that corresponds to cells synthesizing DNA from $G_1$ to $G_2$ (2N → 4N) and (4N → 8N). Results are the mean ± s.d. of four experimental observations. *$P$ <0.001. [a]Fetal liver cells were obtained from fetuses after 22 days of gestation. [b]Time 0 refers to untreated 2-months-old rats (180–200 g).

© Cancer Research Campaign 1997

increased markedly. The extent of hepatocyte population involved in DNA synthesis was significantly enhanced from 30 days; particularly remarkable were the values obtained at 30 days, in which the population of hepatocytes that underwent DNA synthesis was increased almost fourfold. These peaks of DNA replication coincide with peaks of necrosis described in previous studies (Sanz et al, 1995). The population in the S-phase in samples obtained from controls showed a slight increase which may be due to the stress produced by the intraperitoneal injection three times per week of 0.9% sodium chloride.

### DISCUSSION

Chronic liver disease due to cirrhosis is usually accompanied by metabolic alterations affecting energy homeostasis (Nozu et al, 1992). Thioacetamide chronic administration to rats induced the expression of G6PD and ME in the liver throughout the 6 months' treatment. Parallel increases in both specific activity and mRNA of ME and G6PDH were observed. The higher expression of G6PD mRNA in comparison to that of ME may be a consequence of the dual role played by G6PDH in providing both reducing equivalents for detoxifying microsomal oxidation and pentose phosphate for DNA synthesis and repair.

The continuous treatment with thioacetamide produced an imbalance in the redox state of the cell (Tsukamoto et al, 1990), which induced lipid peroxidation. Our data demonstrate that the concentration of hepatic MDA, a metabolite generated in lipoperoxide degradation, was significantly enhanced. Therefore, a close alliance can be observed between the extent of intracellular MDA level and the induction of G6PDH and ME enzyme activities.

The lack of parallelism between the in vivo lipogenesis (Martín-Sanz et al, 1989*a*) and the remarkable induction of ME gene expression in liver from our chronic experimental model of thioacetamide-induced cirrhosis suggests that ME activity is involved in cellular processes other than lipogenesis. It has been shown (Yoshimoto et al, 1983) that ME gene expression in primary cultures of adult hepatocytes does not respond to the mitogenic effects of growth factors (EGF), which seems to indicate that ME activity is not involved in the mechanisms responsible for DNA replication. Thus, the progressive increase in ME mRNA found in our experiments may be due to an involvement of this enzyme in the microsomal biotransformation of drugs and the induction of its expression may be, therefore, a consequence of the cell requirement of reducing power against the oxidative stress. The increased expression of mRNA ME may also be relevant in producing pyruvate, which can be required in chronic diseases and in tumour cells (Loeber et al, 1994).

There are several reports (Lorenzo et al., 1989; Iritani, 1992; Tomita et al, 1993; García-Jinénez et al, 1994) demonstrating that ME acts mostly in lipogenesis, whereas G6PDH functions both in cell growth and in lipogenesis (Yoshimoto et al, 1983). Under proliferative conditions, both in fetal hepatocytes (Molero et al, 1994) and in primary cultures of mature liver cells, it has been shown that the expression of G6PD is markedly induced without changes in ME expression (Stanton et al, 1991). These data, together with those obtained in the present study, suggest that the glucose channelled into fatty acid and pyruvate synthesis decreased in rat liver during the development of liver cirrhosis, while the glucose channelled to pentose phosphates and reducing equivalents increased to favour DNA synthesis and repair and detoxification reactions. Thus, it is proposed that increases in DNA synthesis in liver of rats with hypertrophic macronodular cirrhosis induced experimentally are parallel to a channelling of glucose towards NADPH and pentose phosphate generation and depend on the expression of G6PD. Increases in G6PDH have been reported in preneoplastic and experimental lesions in rat liver induced experimentally. An increased use of NADPH decreases the NADPH/NADP ratio, accelerating carbon flow through the pentose phosphate pathway (Kletzien et al, 1994).

We can conclude from our experiments that the quantitative differential increase in the activity of these two enzymes could be a consequence of the dual role played by G6PDH either providing NADPH for microsomal detoxifying mechanisms or providing ribose 5-phosphate for DNA synthesis and repair, while ME is mainly involved in detoxification. Furthermore, our results show that the oxidant stress due to the oxidative biotransformation of thioacetamide and detected by the levels of MDA are in agreement with the increased expression both of G6PD and ME. The response of each of these two enzymes to the oxidative stress is parallel to their relative rates of gene expression as there is a close relationship between the elevation in the enzyme activities and the amount of mRNA of each enzyme.

In summary, the present results suggest the involvement of G6PDH and ME in the mechanisms of hepatic detoxification. The extent of the contribution of these enzymes to xenobiotic biotransformation is as yet unknown, but our results provide further information regarding NADPH-generating systems which seem to display a gene adaptative response to the oxidative stress in the liver.

### ACKNOWLEDGEMENTS

We thank Mrs Dolores Velasco for her technical assistance and Professor Erik Lundin for his help in the preparation of this manuscript. This work was supported by a grant from Fondo de Investigación Sanitaria (FIS), project no. 95/0032/01.

### REFERENCES

Amasino RM (1986) Acceleration of nucleic acid hybridization rate by polyethylene glycol. *Anal Biochem* **152**: 304–307

Bautista AP and Spitzer JJ (1992) Acute ethanol intoxication stimulates superoxide anion production by in situ perfused rat liver. *Hepatology* **15**: 892–898

Becker FF (1983) Thioacetamide hepatocarcinogenesis. *J Natl Cancer Inst* **71**: 553–558

Bradford MM (1976) A rapid and sensitive method for the quantitation of microgram quantities of protein utilizing the principle of protein-dye-binding. *Anal Biochem* **72**: 248–254

Cascales M, Martín-Sanz P, Craciunescu DG, Mayo I, Aguiliar A, Robles-Chillide E and Cascales C (1991) Alterations in hepatic peroxidation mechanisms in thioacetamide-induced tumors in rats. *Carcinogenesis* **12**: 233–240

Cascales M, Martín-Sanz P, Alvarez A, Sánchez-Pérez M, Díez-Fernández C and Boscá L (1992) Isoenzyme of carbohydrate metabolism in primary cultures of hepatocytes from thioacetamide-induced rat liver necrosis. *Hepatology* **16**: 232–240

Cerdán S, Cascales M and Santos Ruiz A (1981) Effect of thioacetamide on the pentose phosphate pathway and other NADP-linked enzymes of rat liver cytosol. Chronology of perturbations and metabolic significance. *Mol Pharmacol* **19**: 451–455

Cramer CT, Ginsberg LC, Stapleton SR, Kletzien RF and Ulrich RG (1993) Induction of G6PDH in rat hepatocytes under oxidative stress conditions. *Toxicologist* **13**: 20

Chomezynski K and Sacchi N (1987) Single-step method of RNA isolation by acid guanidinium thyocyanate-phenol-chloroform extraction. *Anal Biochem* **162**: 156–159

Desmet VJ (1992) Modulation of the liver in cholestasis. *J Gastroenterol Hepatol* **7**: 313–323

© Cancer Research Campaign 1997 

Deutsch J (1987) Glucose-6-phosphate dehydrogenase. D-Glucose-6-phosphate: $NADP^+$ 1-oxidoreductase EC 1.1.1.49. In *Methods of Enzimatic Analysis*, Vol. III, 3rd edn, Bergmeyer HU. (ed.), pp. 190–197. Verlag Chemie: Weinheim.

Diez-Fernandez C, Boscá L, Fernández-Simón L, Alvarez A and Cascales M (1993) Relationship between genomic DNA ploidy and parameters of liver damage during necrosis and regeneration induced by thioacetamide. *Hepatology* **18**: 912–918

Dozin B, Magnuson MA and Nikodem VM (1985) Tissue-specific regulation of two functional malic enzyme mRNAs triiodothyronine. *Biochemistry* **24**: 5581–5586

Farber E (1990) Clonal adaptation during carcinogenesis. *Biochem Pharmacol* **39**: 1837–1846

García-Jiménez C, Benito B, Jolin T and Santisteban P (1994) Insulin regulation of malic enzyme gene expression in rat liver: evidence for nuclear proteins that bind to two putative insulin response elements. *Mol Endocrinol* **8**: 1361–1369

HO YS, Howard AJ and Crapo JD (1988) Cloning and sequence of a cDNA encoding rat G6PD. *Nucleic acids Res* **16**: 7746

Iritani N (1992) Nutritional and hormonal regulation of lipogenic-enzyme gene expression in rat liver. *Eur J Biochem* **205**: 433–442

Katsurada A, Iritani N, Fukuda H, Noguchi T and Tanaka T (1987) Influence of diet on the transcriptional and post-transcriptional regulation of malic enzyme induction in rat liver. *J Biol Chem* **168**: 487–491

Katsurada A, Iritani N, Fukuda H, Matsumura Y, Noguchi T and Tanaka T (1989) Effects of nutrients and insulin on transcriptional and post-transcriptional regulation of glucose-6-phosphate dehydrogenase synthesis in rat liver. *Biochem Biophys Acta* **1006**: 104–110

Kletzien RF and Berdainer CD (1993) Glucose-6-phosphate dehydrogenase: diet and hormonal influences on de novo enzyme synthesis. In *Nutrition and Gene Expression*, Berdainer CD and Hargrove JL. (eds), pp. 187–206. CRD Press: Boca Ratón.

Kletzien RF, Harris PKW and Foellmi LA (1994) Glucose-6-phosphate dehydrogenase: a 'housekeeping' enzyme subject to tissue-specific regulation by hormones, nutrients, and oxidant stress. *Faseb J* **8**: 174–181

Loeber G, Dworkin MB, Infante A and Ahorn H (1994) Characterization of cytosolic malic enzyme in human tumor cells. *Febs Lett* **344**: 181–186

Lorenzo M, Fabregat I and Benito M (1989) Hormonal regulation of malic enzyme expression in primary cultures of foetal brown adipocytes. *Biochem Biophys Res Commun* **163**: 341–347

Luna LG (1968) Manual of histological staining. In *American Registry of Pathology*. Methods of the Armed Forces Institute of Pathology, pp. 94–98. McGraw-Hill: New York.

Magnuson MA and Nikodem VM (1983) Molecular cloning of a cDNA sequence for rat malic enzyme. *J Biol Chem* **258**: 12712–12717

Martín-Sanz P, Cascales C and Cascales M (1989*a*) Lipogenesis and cholesterogenesis *de novo* in liver and adipose tissue. Alterations of lipid metabolism by the effect of short- and long-term thioacetamide administration to rats. *Carcinogenesis* **10**: 477–481

Martín-Sanz P, Cascales M and Boscá L (1989*b*). Glucagon-induced changes in fructose 2, 6-bisphosphate and 6-phosphofructo-2-kinase in cultured fetal hepatocytes. *Biochem J* **257**: 795–799

Molero C, Benito M and Lorenzo M (1994) Glucose-6-phosphate dehydrogenase gene expression in fetal hepatocyte primary cultures under non-proliferative and proliferative conditions. *Exp Cell Res* **210**: 26–32

Muller D, Sommer M, Kretschmar M, Zimmermann T and Buko VU (1991) Lipid peroxidation in thioacetamide-induced macronodular rat liver cirrhosis. *Arch Toxicol* **65**: 199–203

Niehaus WG, Samuelsson JR and Willis ED (1969) Lipid peroxide formation in microsomes. *Biochem J* **113**: 315–341

Nozu F, Takeyama N and Tanaka T (1992) Changes of hepatic fatty acid metabolism produced by chronic thioacetamide administration in rats. *Hepatology* **15**: 1099–1106

Outlaw Jr WH and Springer SA (1987) Malic enzyme. L-malate: $NAD^+$ oxidoreductase (decarboxylating), EC 1.1.1.39. In *Methods of Enzymatic Analysis*, Vol. III, 3rd edn, Bergmeyer HU. (ed.), pp. 176–183. Verlag Chemie: Weinheim.

Pitot HC (1990) Altered hepatic foci: The role in murine hepatocarcinogenesis. *Annu Rev Pharmacol Toxicol* **30**: 465–500

Sanz N, Díez-Fernández C, Fernández-Simón L, Alvarez A and Cascales M (1995) Relationship between antioxidant systems, intracellular thiols and DNA ploidy in liver of rats during experimental cirrhogenesis. *Carcinogenesis* **16**: 1585–1593

Stanton RC, Sciffer JL, Boxer DC, Zimmermann E and Cantlet LC (1991) Rapid release of bound glucose-6-phosphate dehydrogenase by growth factors. *J Biol Chem* **266** 12442–12448

Tomita Y, Abraham S, Noda C and Ichiara A (1993) Pyruvate stimulates hormonal induction of lipogenic enzymes in primary cultured rat hepatocytes. *Biochem Biophys Acta* **1170**: 253–257

Tsukamoto H, Matsuoka M and French SW (1990) Experimental models of hepatic fibrosis. *Semin Liver Dis* **10**: 56–65

Vindelov LL, Christensen IJ and Nissen NI (1983) A detergent trypsin method for the preparation of nuclei for flow cytometric. *Cytometry* **3**: 323–327

Yoshimoto K, Makamura T and Ichiara A (1983) Reciprocal effects of epidermal growth factor on key lipogenic enzymes in primary cultures of adult rat hepatocytes. Induction of glucose-6-phosphate dehydrogenase and supression of malic enzyme and lipogenesis. *J Biol Chem* **258**: 12355–12360

 © Cancer Research Campaign 1997


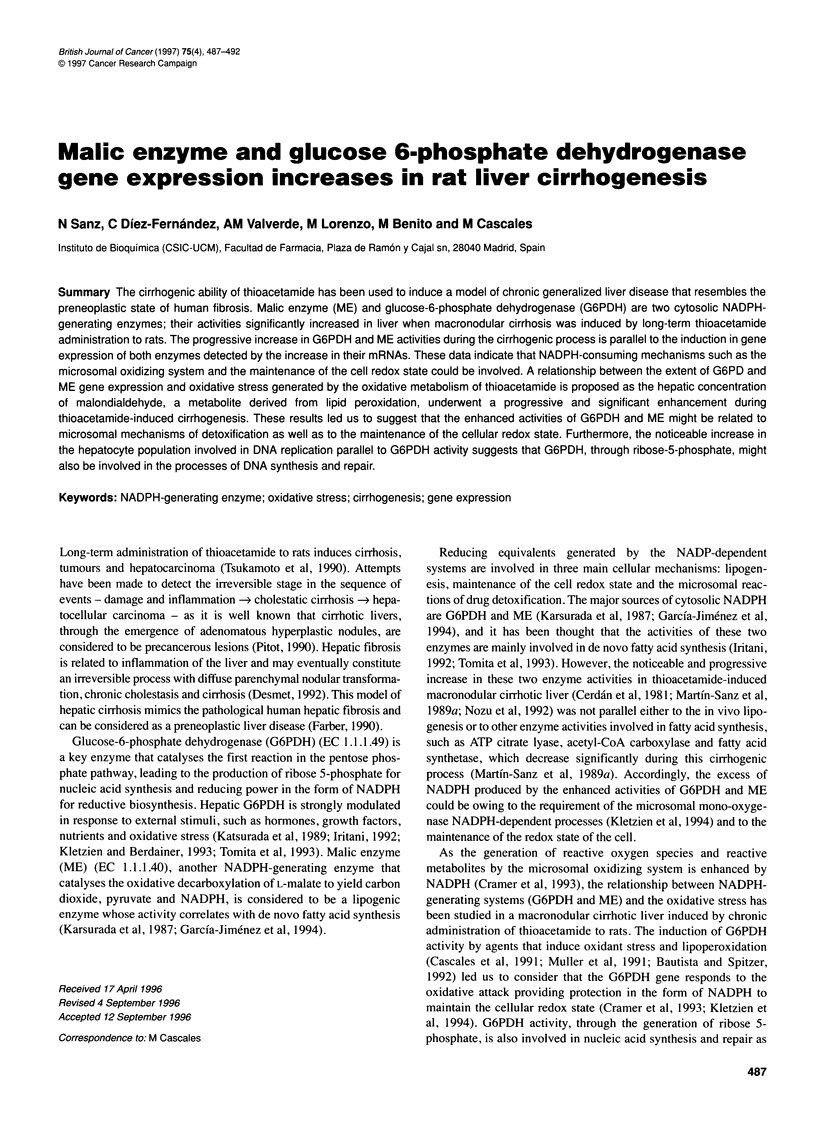

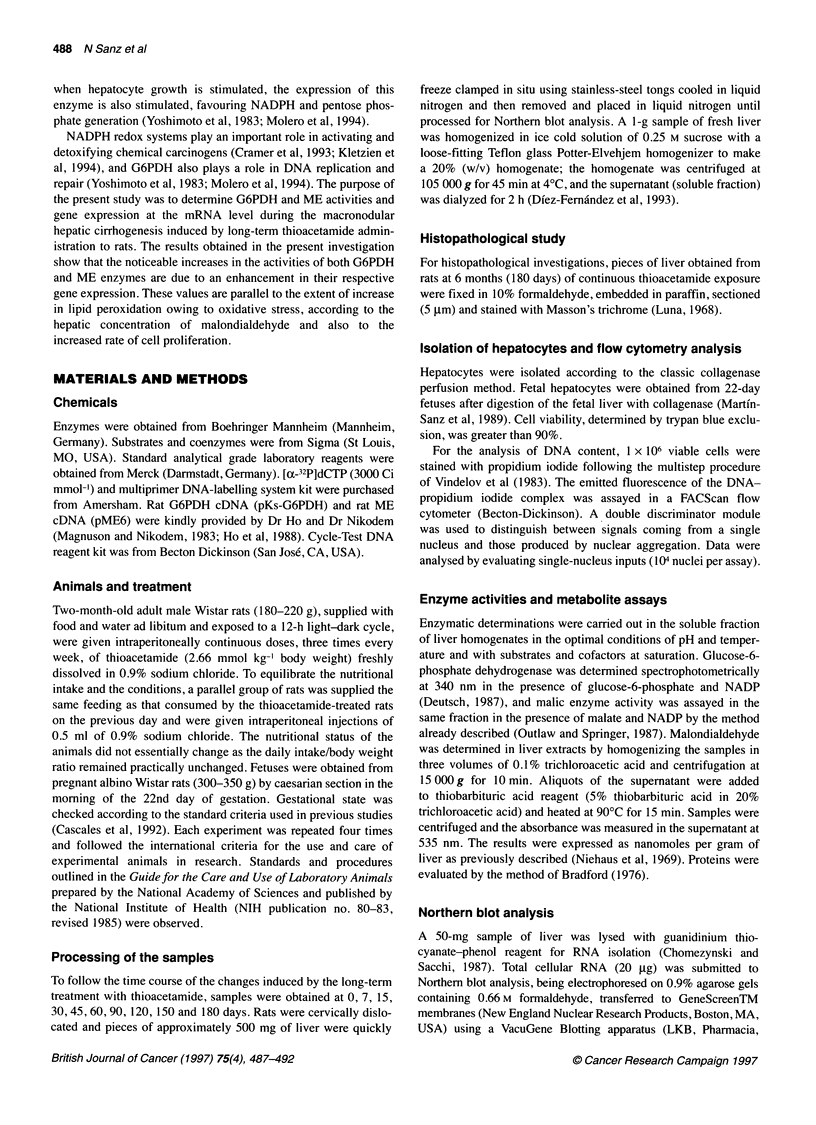

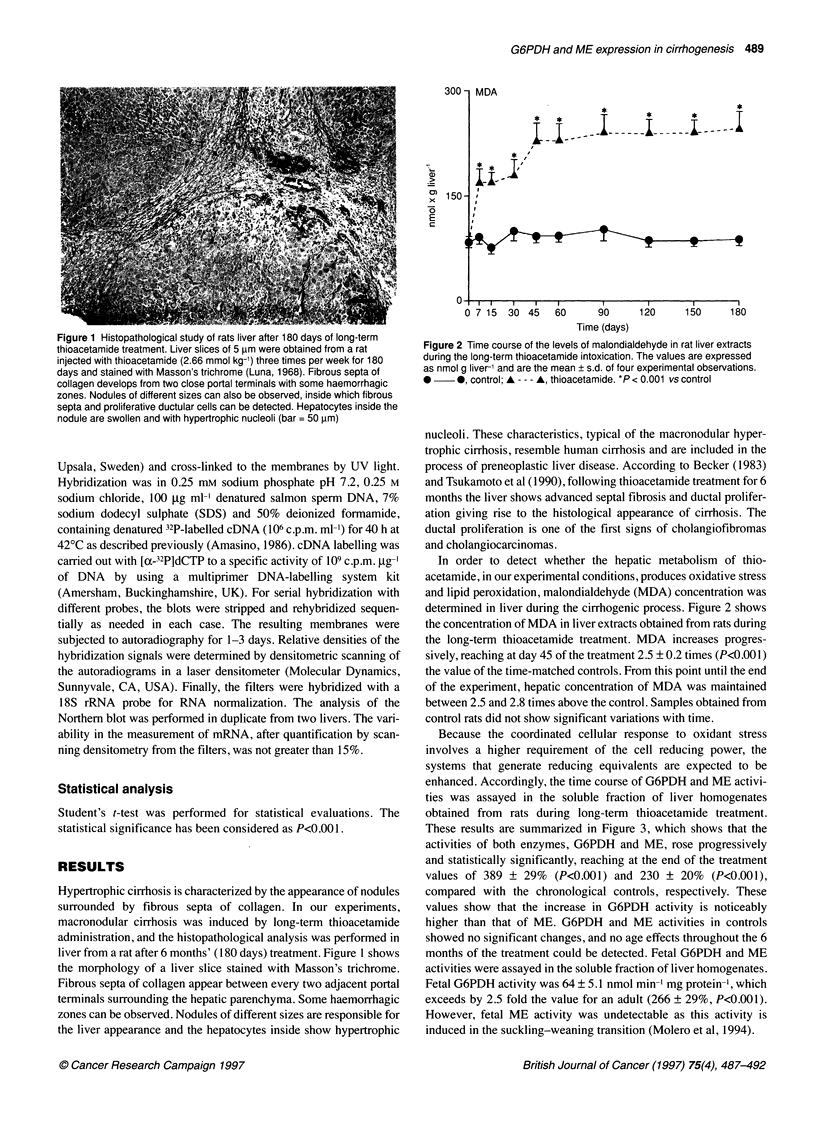

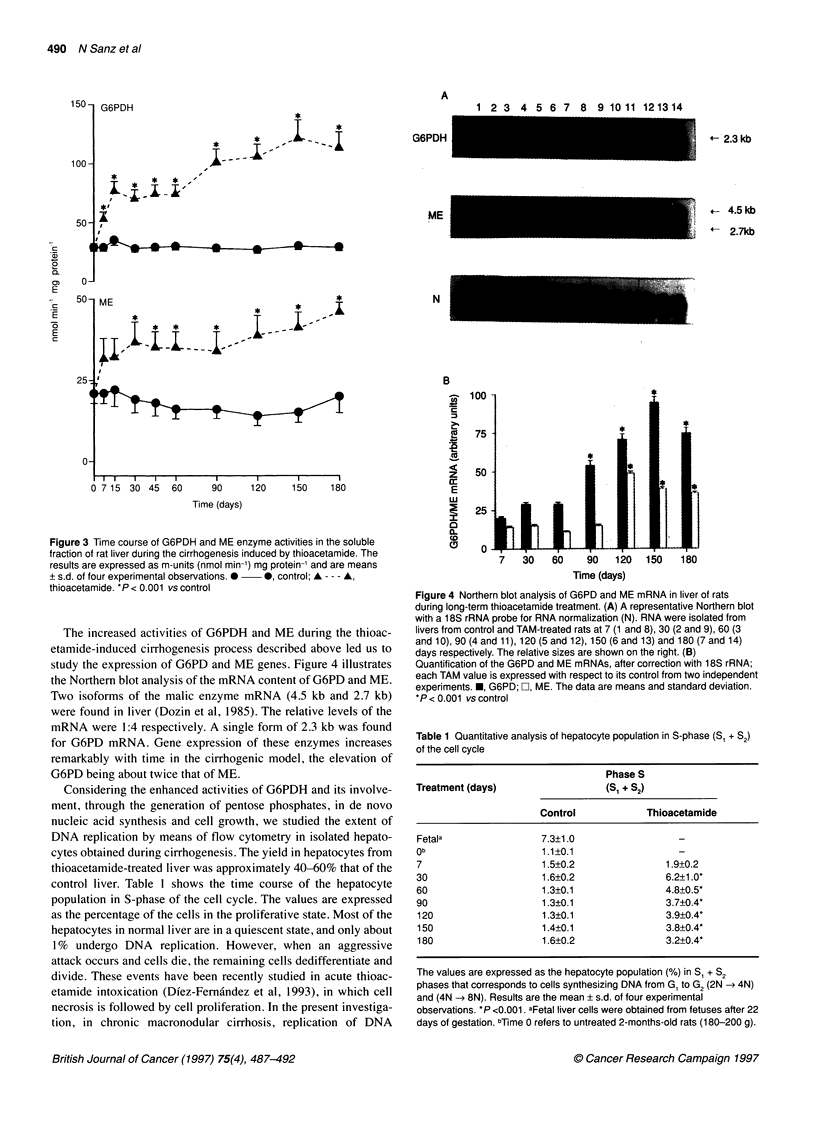

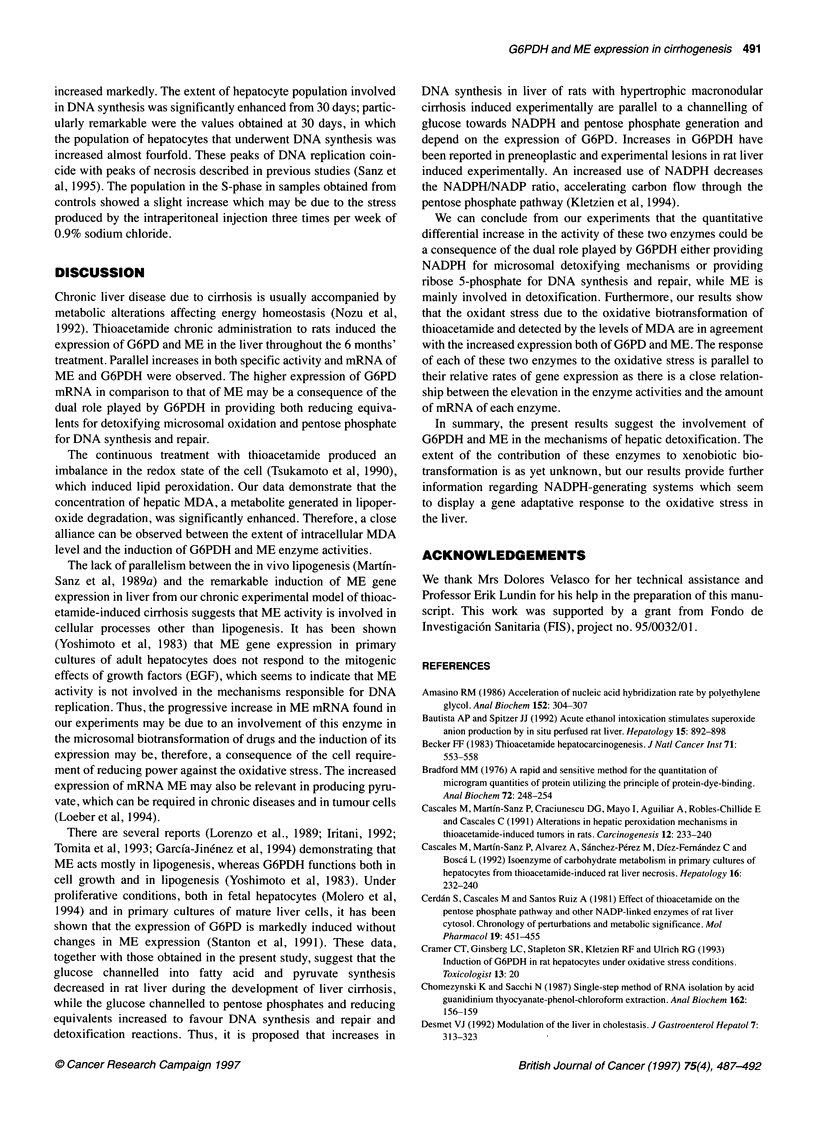

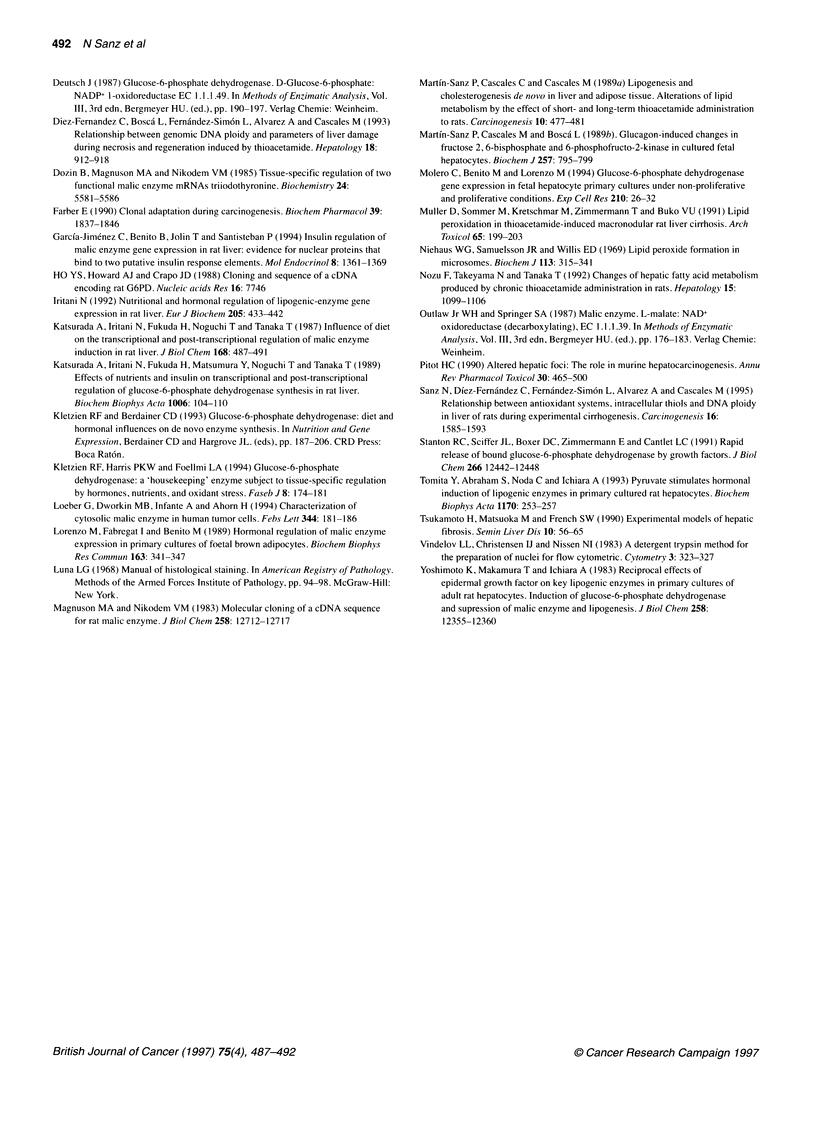


## References

[OCR_00631] Amasino R. M. (1986). Acceleration of nucleic acid hybridization rate by polyethylene glycol.. Anal Biochem.

[OCR_00635] Bautista A. P., Spitzer J. J. (1992). Acute ethanol intoxication stimulates superoxide anion production by in situ perfused rat liver.. Hepatology.

[OCR_00639] Becker F. F. (1983). Thioacetamide hepatocarcinogenesis.. J Natl Cancer Inst.

[OCR_00643] Bradford M. M. (1976). A rapid and sensitive method for the quantitation of microgram quantities of protein utilizing the principle of protein-dye binding.. Anal Biochem.

[OCR_00653] Cascales M., Martin-Sanz P., Alvarez A., Sanchez-Pérez M., Diez Fernández C., Boscá L. (1992). Isoenzymes of carbohydrate metabolism in primary cultures of hepatocytes from thioacetamide-induced rat liver necrosis: responses to growth factors.. Hepatology.

[OCR_00648] Cascales M., Martín-Sanz P., Craciunescu D. G., Mayo I., Aguilar A., Robles-Chillida E. M., Cascales C. (1991). Alterations in hepatic peroxidation mechanisms in thioacetamide-induced tumors in rats. Effect of a rhodium(III) complex.. Carcinogenesis.

[OCR_00659] Cerdán S., Cascales M., Santos-Ruiz A. (1981). Effect of thioacetamide on the pentose phosphate pathway and other NADP-linked enzymes of rat liver cytosol: chronology of the perturbations and metabolic significance.. Mol Pharmacol.

[OCR_00670] Chomczynski P., Sacchi N. (1987). Single-step method of RNA isolation by acid guanidinium thiocyanate-phenol-chloroform extraction.. Anal Biochem.

[OCR_00675] Desmet V. J. (1992). Modulation of the liver in cholestasis.. J Gastroenterol Hepatol.

[OCR_00694] Dozin B., Magnuson M. A., Nikodem V. M. (1985). Tissue-specific regulation of two functional malic enzyme mRNAs by triiodothyronine.. Biochemistry.

[OCR_00688] Díez-Fernández C., Boscá L., Fernández-Simón L., Alvarez A., Cascales M. (1993). Relationship between genomic DNA ploidy and parameters of liver damage during necrosis and regeneration induced by thioacetamide.. Hepatology.

[OCR_00699] Farber E. (1990). Clonal adaptation during carcinogenesis.. Biochem Pharmacol.

[OCR_00703] García-Jiménez C., Benito B., Jolin T., Santisteban P. (1994). Insulin regulation of malic enzyme gene expression in rat liver: evidence for nuclear proteins that bind to two putative insulin response elements.. Mol Endocrinol.

[OCR_00721] Katsurada A., Iritani N., Fukuda H., Matsumura Y., Noguchi T., Tanaka T. (1989). Effects of nutrients and insulin on transcriptional and post-transcriptional regulation of glucose-6-phosphate dehydrogenase synthesis in rat liver.. Biochim Biophys Acta.

[OCR_00716] Katsurada A., Iritani N., Fukuda H., Noguchi T., Tanaka T. (1987). Influence of diet on the transcriptional and post-transcriptional regulation of malic enzyme induction in the rat liver.. Eur J Biochem.

[OCR_00734] Kletzien R. F., Harris P. K., Foellmi L. A. (1994). Glucose-6-phosphate dehydrogenase: a "housekeeping" enzyme subject to tissue-specific regulation by hormones, nutrients, and oxidant stress.. FASEB J.

[OCR_00739] Loeber G., Dworkin M. B., Infante A., Ahorn H. (1994). Characterization of cytosolic malic enzyme in human tumor cells.. FEBS Lett.

[OCR_00743] Lorenzo M., Fabregat I., Benito M. (1989). Hormonal regulation of malic enzyme expression in primary cultures of foetal brown adipocytes.. Biochem Biophys Res Commun.

[OCR_00753] Magnuson M. A., Nikodem V. M. (1983). Molecular cloning of a cDNA sequence for rat malic enzyme. Direct evidence for induction in vivo of rat liver malic enzyme mRNA by thyroid hormone.. J Biol Chem.

[OCR_00757] Martín-Sanz P., Cascales C., Cascales M. (1989). Lipogenesis and cholesterogenesis de novo in liver and adipose tissue. Alterations of lipid metabolism by the effect of short- and long-term thioacetamide administration to rats.. Carcinogenesis.

[OCR_00769] Molero C., Benito M., Lorenzo M. (1994). Glucose-6-phosphate dehydrogenase gene expression in fetal hepatocyte primary cultures under nonproliferative and proliferative conditions.. Exp Cell Res.

[OCR_00783] Nozu F., Takeyama N., Tanaka T. (1992). Changes of hepatic fatty acid metabolism produced by chronic thioacetamide administration in rats.. Hepatology.

[OCR_00795] Pitot H. C. (1990). Altered hepatic foci: their role in murine hepatocarcinogenesis.. Annu Rev Pharmacol Toxicol.

[OCR_00799] Sanz N., Díez-Fernández C., Fernández-Simón L., Alvarez A., Cascales M. (1995). Relationship between antioxidant systems, intracellular thiols and DNA ploidy in liver of rats during experimental cirrhogenesis.. Carcinogenesis.

[OCR_00805] Stanton R. C., Seifter J. L., Boxer D. C., Zimmerman E., Cantley L. C. (1991). Rapid release of bound glucose-6-phosphate dehydrogenase by growth factors. Correlation with increased enzymatic activity.. J Biol Chem.

[OCR_00810] Tomita Y., Abraham S., Noda C., Ichihara A. (1993). Pyruvate stimulates hormonal induction of lipogenic enzymes in primary cultured rat hepatocytes.. Biochim Biophys Acta.

[OCR_00815] Tsukamoto H., Matsuoka M., French S. W. (1990). Experimental models of hepatic fibrosis: a review.. Semin Liver Dis.

[OCR_00819] Vindeløv L. L., Christensen I. J., Nissen N. I. (1983). A detergent-trypsin method for the preparation of nuclei for flow cytometric DNA analysis.. Cytometry.

[OCR_00779] Wills E. D. (1969). Lipid peroxide formation in microsomes. General considerations.. Biochem J.

[OCR_00822] Yoshimoto K., Nakamura T., Ichihara A. (1983). Reciprocal effects of epidermal growth factor on key lipogenic enzymes in primary cultures of adult rat hepatocytes. Induction of glucose-6-phosphate dehydrogenase and suppression of malic enzyme and lipogenesis.. J Biol Chem.

